# Micro-Slab Coil Design for Hyperpolarized Metabolic Flux Analysis in Multiple Samples

**DOI:** 10.3390/bioengineering10010014

**Published:** 2022-12-21

**Authors:** Geonhui Lee, Thomas Ruan, Claudia Wong, Kofi Deh, Alli Abolarin, Alexander Correa, Kayvan R. Keshari, Sangmoo Jeong

**Affiliations:** 1 Department of Chemical and Biomolecular Engineering, Johns Hopkins University, Baltimore, MD 21218, USA; 2 Institute for NanoBioTechnology, Johns Hopkins University, Baltimore, MD 21218, USA; 3 Department of Radiology, Memorial Sloan Kettering Cancer Center, New York, NY 10065, USA; 4 Molecular Pharmacology Program, Memorial Sloan Kettering Cancer Center, New York, NY 10065, USA; 5 Physiology, Biophysics and Systems Biology Program, Weill Cornell Medical College, New York, NY 10065, USA

**Keywords:** hyperpolarization, 13C-magnetic resonance, metabolic flux, micro-NMR coil

## Abstract

Abnormal metabolism is a hallmark of cancer cells. Accumulating evidence suggests that metabolic changes are likely to occur before other cellular responses in cancer cells upon drug treatment. Therefore, the metabolic activity or flux in cancer cells could be a potent biomarker for cancer detection and treatment monitoring. Magnetic resonance (MR)-based sensing technologies have been developed with hyperpolarized molecules for real-time flux analysis, but they still suffer from low sensitivity and throughput. To address this limitation, we have developed an innovative miniaturized MR coil, termed micro-slab MR coil, for simultaneous analysis of metabolic flux in multiple samples. Combining this approach with hyperpolarized probes, we were able to quantify the pyruvate-to-lactate flux in two different leukemic cell lines in a non-destructive manner, simultaneously. Further, we were able to rapidly assess flux changes with drug treatment in a single hyperpolarization experiment. This new multi-sample system has the potential to transform our ability to assess metabolic dynamics at scale.

## 1. Introduction

Abnormal metabolism has been implicated in various diseases, including cancer [1,2,3]. For example, a significantly higher rate of glycolysis, commonly driven by oncogenic mutations such as PI3K and MYC, is required for cancer cells to meet their energetic and anabolic needs [4,5]. Furthermore, dysfunctional metabolic enzymes can lead to oncogenesis. Mutations in isocitrate dehydrogenase 1 and 2 (IDH1 and IDH2, respectively) generate 2-hydroxyglutarate (2-HG), disrupting epigenetic regulations [6,7]. Therefore, metabolism can be not only the consequence of oncogenesis but also the cause.

The abnormal metabolic features of malignant cells have been exploited for detection and treatment modalities [8]. The increased uptake of glucose by cancer cells is the underlying mechanism of fluorodeoxyglucose-positron emission tomography (FDG-PET), one of the most widely used imaging methods for cancer diagnosis. Also, magnetic resonance spectroscopy (MRS) has been developed to investigate metabolism in vivo without a limitation of detection depth. For example, ^1^H-MRS techniques enabled in vivo detection of 2-HG in glioblastoma patients with IDH1 mutation [9,10]. In addition, ^13^C- or ^2^H-MRS techniques enabled more targeted analyses in specific metabolic pathways with an infusion of ^13^C- or ^2^H-enriched metabolites, including [U-^13^C] glucose [11] and [6,6′-^2^H_2_] glucose [12]. Recently, ^13^C-MRS methods have been developed in conjunction with hyperpolarization techniques to quantify metabolic fluxes in real-time [13,14]. Since magnetic resonance signal is determined by the spin polarization level (a population difference between high- and low-spin states), a hyperpolarization approach can increase MR signal by a factor of >10,000, enabling previously unattainable investigations of metabolic fluxes [13]. In particular, the dissolution dynamic nuclear polarization (dDNP) method has been applied to various preclinical and clinical applications with hyperpolarized ^13^C-enriched metabolites, including [1-^13^C] pyruvate [15,16,17], [6-^13^C, ^15^N_3_] arginine [18], [1-^13^C] lactate [19,20], and [1-^13^C] glutamine [21].

We previously demonstrated an integrated platform that took advantage of dDNP and a miniaturized detection coil for sensitive analysis of pyruvate-to-lactate flux in mass-limited samples; termed hyperpolarized micro-magnetic resonance spectrometer [22]. This platform enabled non-destructive quantification of metabolic flux in a small number of living cells, on the order of 10^4^ cells. While this miniaturized coil achieved an unprecedented sensitivity for the flux analysis, its single channel format limited the analytical throughput. Since a hyperpolarized state decays exponentially with a spin-lattice relaxation time T_1_ (~1 min for [1-^13^C] pyruvate) [23], it was challenging to analyze the metabolic flux in different samples from a single dDNP experiment. While we have devised methods to increase the lifetime of hyperpolarized probes, enabling a higher analytical throughput [18,24], the single channel platform requires the measurement of multiple samples sequentially [25]. Here, we report a novel miniaturized coil design, termed a micro-slab coil, for simultaneous analysis of metabolic flux in multiple samples. With this design, a coil can be positioned close to all the samples uniformly. Therefore, sensitive acquisition of magnetic resonance (MR) signal from multiple samples can be achievable. As a proof-of-concept, we developed a three-channel micro-slab coil system with a 3D-printed structure and demonstrated the flux difference in two cancer cell lines and the flux change upon drug treatment. Our micro-slab coil can provide a higher throughput and sensitivity than conventional coils and can be applicable for various MR experiments. Given that a hyperpolarization method (e.g., dDNP) requires an hour-long preparation step and expensive reagents, our design will be significantly beneficial for metabolic flux analysis with hyperpolarized probes.

## 2. Materials and Methods

### 2.1. Hyperpolarization Experiments

We used the dDNP method to hyperpolarize [1-^13^C] pyruvate. The following describes the sample preparation steps: (1) [1-^13^C] pyruvic acid (Sigma-Aldrich, St. Louis, MO, USA) was thoroughly mixed with 15 mM AH-111501 (GE Healthcare, Chicago, IL, USA), 10 μL of which was loaded into the SPINlab polarizer (5 T, 0.87 K, GE Healthcare, Chicago, IL, USA). (2) 100 mM Trizma hydrochloride solution (Sigma-Aldrich, St. Louis, MO, USA) with 1 mM ethylenediaminetetraacetic acid (EDTA) (Sigma-Aldrich, St. Louis, MO, USA) was prepared in deuterium oxide (D_2_O) as dissolution buffer solution [24]. (3) After 120-min polarization, the pyruvate sample was quickly dissolved into a flask and neutralized with 13 μL of 10 N sodium hydroxide solution (Fisher Scientific, Waltham, MA, USA). The sample was then mixed with cell suspension, which was prepared right before the dissolution, with a ratio of 1 to 4 (10 μL pyruvate sample and 40 μL cell suspension); this made the final concentration of [1-^13^C] pyruvate approximately 5 mM.

### 2.2. Acquisition and Processing of MR Signals

The micro-slab coil circuit was designed to have two channels, one for ^1^H-MR and the other for ^13^C-MR, which were directly connected to a preclinical MRI system (BioSpec 3T, Bruker, Billerica, MA, USA). For ^1^H-MR signal acquisition, a segmented fast low-angle shot (SegFLASH) sequence was used with the following parameters: 40 mm field of view, 3 mm slice thickness, 256 × 256 matrix size, a repetition time (TR) of 60 ms, an echo time (TE) of 5.248 ms, and a radio-frequency pulse of 30° flip angle. For hyperpolarized ^13^C-MR signal acquisition, a one-dimensional chemical shift imaging (CSI) sequence was used to localize signal from the three wells. Nine voxels with 2.22 mm resolution were proscribed in the direction of the magnet bore, forming a 20 mm field of view covering the length of the micro-slab. No slice gradient was used. The scanner was set to continuously acquire signal throughout the experiment, with the following acquisition parameters: a TR of 11.111 ms (one 9-voxel image per second), a TE of 0.568 ms, a radio-frequency pulse of 10° flip angle (30° per image), a bandwidth of 155.8 ppm (512 points). The acquired free induction decay data were zero-filled once and multiplied by an exponential window function of 5 Hz before Fourier transform. The transformed spectra were phased and baseline-corrected by voxel in the software MestReNova (Mestrelab Research, Santiago de Compostela, Spain), followed by the integration of NMR peak areas; specifically, we focused on MR peaks of [1-^13^C] pyruvate, [1-^13^C] pyruvate hydrate, and [1-^13^C] lactate. The pyruvate-to-lactate conversion rate was calculated as described in our previous studies [22,25]; the rate constant k_PL_ (s^−1^) was represented by the linear change of ^13^C-lactate signal (integral of [1-^13^C] lactate peak) relative to ^13^C-total signal (integral sum of [1-^13^C] pyruvate, [1-^13^C] pyruvate hydrate, and [1-^13^C] lactate peaks), and the flux metric ξ (pmol/s/10^5^ cells) was represented by the product of k_PL_ and the initial concentration of [1-^13^C] pyruvate normalized by cell number.

### 2.3. Calculation of the Average Distance between the Micro-Solenoid Coil and Origin of Samples

To calculate the average distance between the micro-solenoid and origin of samples, we first determined the distance between each sample and micro-solenoid. For a single sample, the distance between the sample circle and micro-solenoid is $\frac{1}{10}R_{1}$. For multiple samples, the shortest distances between all samples and micro-solenoid are $\frac{1}{10}R_{1}$. We then determined the average distance between the micro-solenoid and origin of samples:

(1) $$D_{avg}=\frac{1}{2\pi }\int_{0}^{2\pi }\sqrt{{\left(R_{1}cos\theta -h\right)}^{2}+R_{1}{}^{2}sin^{2}\theta \;}d\theta$$

where $D_{avg}$ is average distance between the micro-solenoid and origin of sample and *h* is the origin of sample (h≥0).

### 2.4. Cell Culture

MOLM13 and NOMO1 were purchased from DSMZ (Braunschweig, Germany). They were cultured in RPMI-1640 medium (Thermo Fisher, Waltham, MA, USA) supplemented with 10% fetal bovine serum (Thermo Fisher, Waltham, MA, USA) and 1% penicillin streptomycin (Thermo Fisher, Waltham, MA, USA) at 37 °C and 5% CO_2_. For the hyperpolarization experiments, the target cells were suspended immediately before the experiment by centrifugation for 3 min at 1000 rpm and resuspension to a volume of 45 μL per replicate.

### 2.5. Analysis of Intracellular Level of NAD+ and NADH

The levels of NAD^+^ and NADH were measured using a commercially available kit NAD/NADH-Glo (Promega, Madison, WI, USA), as described in the manufacturer’s protocol. Briefly, 10^5^ cells of interest were suspended in 50 μL of media in a 96-well plate. Then, 50 μL of cell lysis solution was added into each well. The lysed sample was divided into two wells, one for NAD^+^ measurement and the other for NADH, followed by incubation at 60 °C for 15 min. After cooling the plate at room temperature for 10 min, Trizma base solution was added into the sample well for NAD^+^ measurement, followed by addition of HCl/Trizma base solution into the sample well for NADH measurement. Then, 100 μL of the NAD/NADH-Glo detection reagent was added into each well. After 60-min incubation at room temperature, the luminescence level of each well was measured in a plate reader (Synergy H4 Microplate Reader, BioTek, Winooski, VT, USA).

### 2.6. Analysis of Extracellular Level of Lactate

The level of lactate in cell culture media was measured using a commercially available kit Lactate-Glo (Promega, Madison, WI, USA), as described in the manufacturer’s protocol. Briefly, media samples were collected at experimental time points and diluted with PBS with a ratio of 1:20. The diluted media sample was added into a 96-well plate with the lactate detection reagent in a 1:1 ratio. After 60-min incubation at room temperature, the luminescence level of each well was measured in a plate reader (Synergy H4 Microplate Reader, BioTek, Winooski, VT, USA).

### 2.7. Drug Treatment

ABT199 (Cayman Chemical, Ann Arbor, MI, USA) solution was prepared in dimethyl sulfoxide (DMSO) and added into cell culture media with a dilution factor of 1:1000; specifically, we prepared 10 and 50 μM ABT199 solution and treated the cells with a final concentration of 10 and 50 nM. The viability and cell number were assessed after 24 and 48 h of ABT-199 treatment using Trypan Blue (Sigma-Aldrich, St. Louis, MO, USA). The molecular analyses, including the hyperpolarized pyruvate-to-lactate flux analysis and the measurement of intracellular level of NAD^+^ and NADH, were performed after 24-h treatment.

### 2.8. Immunoblot Analysis

Cell lysates prepared in RIPA buffer (Thermo Fisher, Waltham, MA, USA) were separated in NuPAGE 4–12% Bis-Tris gels (Thermo Fisher, Waltham, MA, USA), and proteins were transferred for two hours at room temperature to PVDF membranes. Membranes were blotted with the following primary antibodies: LDHA antibody (1:1000; #2012s; Cell Signaling Technology, Danvers, MA, USA), phospho-LDHA (1:1000; #8176S; Cell Signaling Technology, Danvers, MA, USA), and β-actin antibody (1:1000; #12620s; Cell Signaling Technology, Danvers, MA, USA). Primary antibodies were detected with HRP-conjugated IgG antibody (1:1000; #32460; Thermo Fisher, Waltham, MA, USA). Pierce ECL Western Blotting Substrate (Thermo Fisher, Waltham, MA, USA) was used to visualize protein bands.

### 2.9. Flow Cytometry Analysis

Cells were washed twice with PBS and incubated in FACS staining buffer (PBS with 2% FBS and 0.5 mM EDTA) along with their respective primary antibody: MCT1 (1:40; #20139-1-AP; Thermo Fisher, Waltham, MA, USA) and MCT4 (1:40; #22787-1-AP; Thermo Fisher, Waltham, MA, USA) for one hour at room temperature. Cells were then washed twice with PBS and incubated in FACS staining buffer with FITC-conjugated IgG secondary antibody (1:80; #F-2765; Thermo Fisher, Waltham, MA, USA) for 1 h at room temperature. Lastly, cells were washed twice with PBS and analyzed using a flow cytometer (BD FACSCanto Clinical Flow Cytometry System, BD Biosciences, Franklin Lakes, NJ, USA).

## 3. Results and Discussion

### 3.1. Micro-Slab Coil Design Achieves a Higher Detection Sensitivity and Generates a More Homogeneous Magnetic Field Than Micro-Solenoid Coil Design

**Figure 1:**
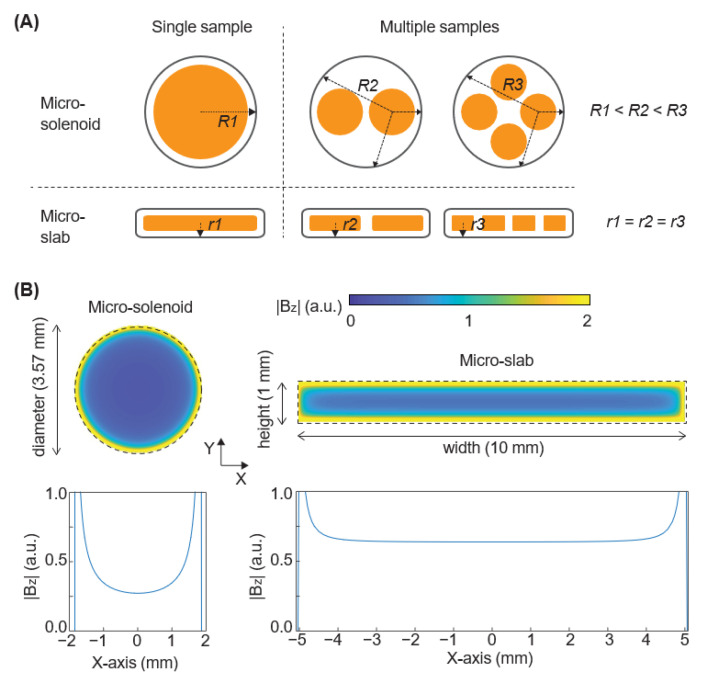
Micro-slab design outperforms micro-solenoid design for magnetic signal detection in multiple samples. (**A**) Schematic comparing the cross-section of a micro-solenoid coil design and a micro-slab coil design. The sample-to-coil distance in the micro-solenoid, *R*, increases with the number of samples inside, but the sample-to-coil distance in the micro-slab, *r*, does not increase. (**B**) Electromagnetic simulation comparing the magnitude and uniformity of the magnetic field (B_z_) inside a micro-solenoid coil and a micro-slab coil. The simulation was performed with the condition that each coil design had the same cross-sectional area.

A miniaturized coil can detect hyperpolarized MR signals more sensitively, mainly due to a shorter sample-to-coil distance [26]. A micro-solenoid coil is one of the most widely used miniaturized coil designs to analyze a small volume of samples in various applications [27,28,29,30]. However, this coil design suffers from an inhomogeneous excitation field due to its small size and shape, which makes it impractical to implement multiple sample inlets inside a single micro-solenoid coil for higher analytical throughput. A homogeneous magnetic field for excitation could be generated by a separate large coil, but the distance from each sample to a micro-solenoid coil would be increased with multiple samples, leading to a lower detection sensitivity (Figure 1A and Figure S1). Therefore, we invented a new micro-coil design for sensitive detection of MR signals from multiple samples. This design is slab-shaped, where individual microwells are positioned along a long axis, and thus all the wells have a uniform sample-to-coil distance (Figure 1A). Also, this ‘micro-slab’ coil design could generate a homogeneous excitation magnetic field over all the samples, another advantage over a micro-solenoid design. We compared the magnetic field generated by the two designs (micro-slab vs micro-solenoid) using electromagnetic simulation with MATLAB (MathWorks, Natick, MA, USA). Two-dimensional analysis of magnetic field generated by the two coils over the same cross-sectional area shows that the micro-slab coil generates an excitation field with less than 10% variation over 24% of the area inside the coil, whereas the micro-solenoid generates a field with the same variation over 12% of the area inside the coil (Figure 1B). In addition, a magnetic field generated by a unit current is higher in the micro-slab coil than in the micro-solenoid, indicating a higher detection sensitivity of the micro-slab coil due to the reciprocal principle. Altogether, these results indicate that our micro-slab coil has advantages over a conventional micro-solenoid coil for accurate and sensitive MR analysis from multiple samples.

### 3.2. Micro-Slab Coil with Three Separate Wells Enables Simultaneous Mr Analysis of Three Different Samples

**Figure 2:**
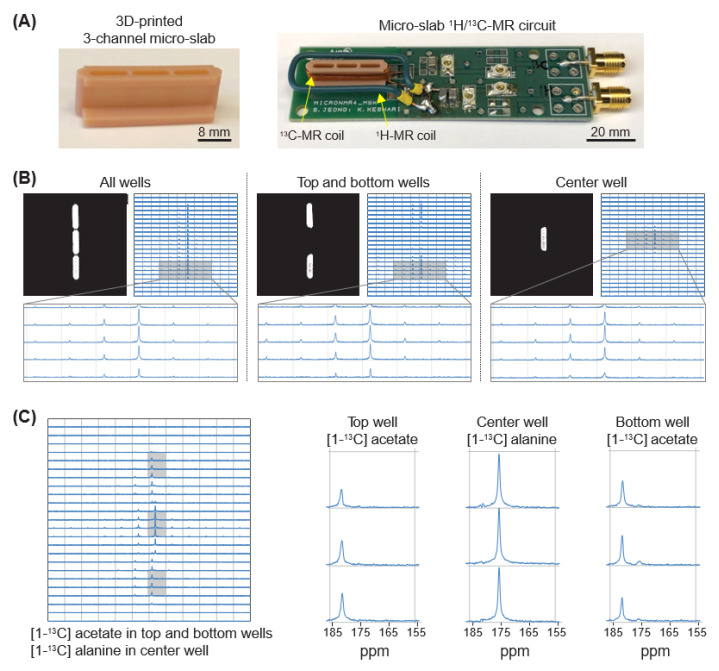
Multi-channel micro-slab MR coil analyzes different samples simultaneously. (**A**) Optical picture of the system. A 3-channel micro-slab construct was made with a 3D printer (**Left**). A 28-gauge magnetic wire was wound around the micro-slab construct and connected to ^13^C-MR circuit in a custom-designed ^1^H/^13^C-MR circuit board (**Right**). A single-loop coil was connected to ^1^H-MR circuit. (**B**) Three sets of a T_2_-weighted MR image and an array of 2D-CSI ^13^C-MR spectra acquired when 4 M [1-^13^C] sodium acetate solution was loaded into wells accordingly. (**C**) 2D-CSI ^13^C-MR spectra acquired when 4 M [1-^13^C] acetate solution was loaded into the top and bottom wells and 8 M [1-^13^C] alanine solution was loaded into the center well.

To implement our novel micro-slab coil, we first designed the micro-slab structure with a three-dimensional (3D) computer-aided design software, 3ds Max (Autodesk, San Rafael, CA, USA), and fabricated it using a 3D printer, Micro Plus HD (EnvisionTec, Gladbeck, Germany). In the micro-slab structure, we designed three separate wells, each of which had a cross section of 2 mm (width) × 8 mm (height) (Figure 2A, Left). A 28-gauge magnetic wire was wound around the 3-well micro-slab structure with a depth of 8 mm, which formed a detection volume of approximately 130 μL for each well. The fabricated micro-slab coil was mounted on a custom-designed printed circuit board equipped with variable capacitors for tuning and matching (Figure 2A, Right). We also added a single-loop ^1^H-MR coil around the 3-well micro-slab construct for shimming and positioning inside an MRI magnet (BioSpec 3T, Bruker, Billerica, MA, USA) based on ^1^H-MR signal. Our printed circuit board had two separate channels, one for ^1^H-MR and another for ^13^C-MR, and thus we could use two different MR modes without a switch [22]. To determine the characteristics of three wells, we loaded 4M [1-^13^C] sodium acetate in each well in the following three different patterns: filling all wells, only top and bottom, and only the center well, and acquired a T_2_-weighted ^1^H-MR image and 2D chemical-shift imaging (CSI) ^13^C-MR spectra (Figure 2B). The ^13^C signals corresponded with the sample location, as seen in the ^1^H-MR images. We then investigated whether the ^13^C-MR signal from the center well would interfere with those from the top and bottom wells by loading 8 M [1-^13^C] alanine solution in the center well and 4 M [1-^13^C] sodium acetate solution in the top and bottom wells (Figure 2C). [1-^13^C] alanine and [1-^13^C] sodium acetate have peaks at 176 and 181.5 ppm, respectively, and they have a high solubility (over 4 M) in water. Therefore, these chemicals could be advantageous to the interference test between wells without hyperpolarization. 2D-CSI ^13^C-MR analysis showed that the interference between adjacent wells would be minimal. Cumulatively, these results demonstrate that our micro-slab coil could be an effective platform for simultaneous MR analysis of multiple samples.

### 3.3. Micro-Slab MR Coil in Conjunction with Hyperpolarized Probes Enables Quantitative Analysis of Metabolic Flux in Different Biological Samples

**Figure 3:**
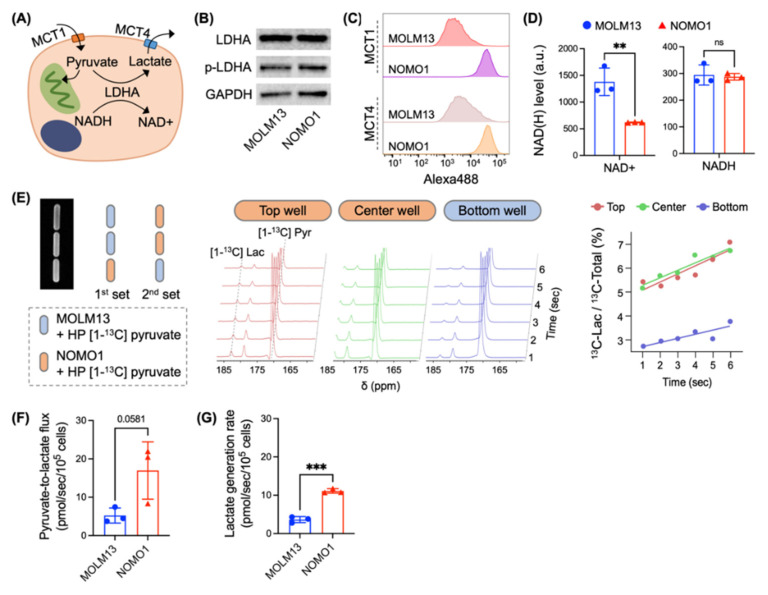
Micro-slab MR coil with hyperpolarized [1-^13^C] pyruvate enables simultaneous assessment of metabolic flux in different living cells. (**A**) Schematic of pyruvate-to-lactate metabolism. Pyruvate is transported into a cell through monocarboxylate transporter 1 (MCT1) and lactate is transported out through MCT4. (**B**) The expression level of LDHA and its phosphorylated form (p-LDHA) in MOLM13 and NOMO1 cells. Original Western blot images are provided in Supplementary Figure S3. (**C**) The surface expression level of MCT1 and MCT4 in MOLM13 and NOMO1 cells. (**D**) Levels of NAD^+^ and NADH in MOLM13 and NOMO1. (**E**) Example dataset of hyperpolarized experiment with MOLM13 and NOMO1 cells in the 3-well micro-slab coil. T_2_-weighted ^1^H-MR image showing filled sample wells and schematic of sample loading procedure (**Left**). Dynamic ^13^C-MR spectra from the 2nd set of samples, with NOMO1 loaded in top and center wells and MOLM13 loaded in bottom well (**Middle**). Graph of [1-^13^C] lactate signal divided by total ^13^C-MR signal against time (**Right**). The slope of a linear fit to these points is normalized by HP [1-^13^C] pyruvate concentration and number of cells to give the pyruvate-to-lactate flux rate. (**F**) Pyruvate-to-lactate flux of MOLM13 and NOMO1 cells measured in the experiment (**E**). (**G**) Lactate generation rate of MOLM13 and NOMO1 cells based on the difference in lactate level in the media and cell number after 24 h of culture. All graphs show mean ± SD (*n* = 3 technical replicates) and are representative of two independent experiments. Statistical analyses were conducted with unpaired two-tailed *t* test: ns (not significant) *p* > 0.05, ** *p* < 0.01, *** *p* < 0.001.

To validate our new coil as a platform for metabolic flux analysis in multiple biological samples, we used the micro-slab coil to measure the metabolic flux of hyperpolarized [1-^13^C] pyruvate in two acute myeloid leukemia cell lines, MOLM13 and NOMO1. These two cell lines have distinct features in pyruvate-to-lactate metabolism (Figure 3A): while the levels of lactate dehydrogenase (LDHA) expression and phosphorylation are similar in both cells, NOMO1 cells has significantly higher expression levels of monocarboxylate transporter (MCT)1 and MCT4 over MOLM13 cells (Figure 3B,C and Figure S2A). This drastic difference in MCT1 and MCT4 levels could be attributed to p53; previous studies showed that the level of p53, a transcriptional repressor of MCT1 and MCT4 [31,32], was significantly higher in MOLM13 cells than in NOMO1 [33,34]. In addition, NAD^+^ level was much lower in NOMO1 than MOLM13 cells, while NADH level was similar in both cells (Figure 3D). Considering that MCT1 and MCT4 are key transporters of pyruvate and lactate and NAD^+^ and NADH are cofactors for LDHA activity (Figure 3A) [16,35], we hypothesized that pyruvate-to-lactate flux would be higher in NOMO1 cells. As the micro-slab coil had three wells, we loaded MOLM13 and NOMO1 cells in two different combinations (Figure 3E). Signals from the individual wells were localized with a 1D-CSI sequence that proscribed voxels along the long dimension of the coil. Voxels representing signal from individual samples were independently analyzed to give hyperpolarized pyruvate-to-lactate flux rates in different samples. As expected, the flux analysis with hyperpolarized [1-^13^C] pyruvate showed that NOMO1 cells exhibited a significantly higher pyruvate-to-lactate flux (16.96 ± 4.31 pmol/s/10^5^ cells) than MOLM13 cells (5.23 ± 1.14 pmol/s/10^5^ cells) (Figure 3F). We also measured the lactate generation rate based on the change in lactate level in the culture media (Figure 3G). Interestingly, the lactate generation rate (11.12 ± 4.31 pmol/s/10^5^ cells for NOMO1; 3.64 ± 0.79 pmol/s/10^5^ cells for MOLM13) was slightly smaller than the pyruvate-to-lactate flux of each cell line measured with hyperpolarized [1-^13^C] pyruvate. The difference between the pyruvate-to-lactate flux (analysis from the hyperpolarized experiment) and the lactate generation rate (analysis from the metabolites in media) could be explained by different numbers of metabolic steps; as lactate in the culture media is mainly derived from glucose through the glycolysis pathway, its generation rate could be limited by glucose transporters or other steps above pyruvate. Still, the difference is marginal, supporting that pyruvate-to-lactate flux has a direct correlation with glycolytic flux [22,36].

### 3.4. Micro-Slab MR Coil with HP [1-^13^C] Pyruvate Enables the Rapid Assessment of Pyruvate-to-Lactate Flux in Cancer Cells Treated with Different Doses of Drug

**Figure 4:**
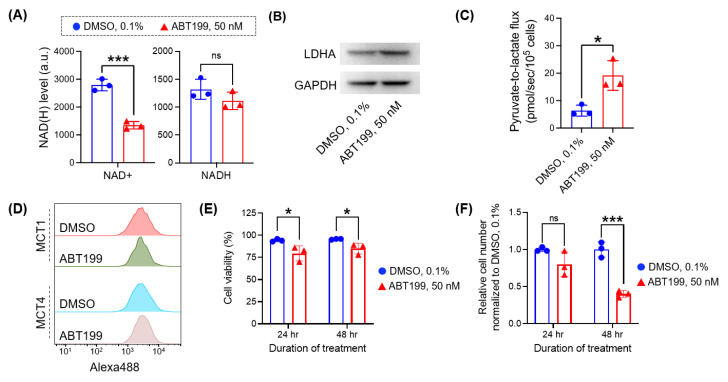
Micro-slab MR coil with HP [1-^13^C] pyruvate enables simultaneous assessment of metabolic flux in cancer cells with different doses of drug treatment. (**A**) Levels of NAD^+^ and NADH in MOLM13 cells after 24-h treatment of ABT199 (mean ± SD, *n* = 3 biological replicate). (**B**) The expression levels of LDHA in MOLM13 cells after 24-h treatment of ABT199. Original Western blot images are provided in Supplementary Figure S3. (**C**) Pyruvate-to-lactate flux in MOLM13 cells after 24-h treatment of ABT199. It was measured with the micro-slab MR coil using hyperpolarized [1-^13^C] pyruvate. (**D**) The expression levels of MCT1 and MCT4 in MOLM13 cells after 24-h treatment of ABT199. (**E**,**F**) Cell number (**E**) and viability (**F**) of MOLM13 cells with ABT199 treatment (mean ± SD, *n* = 3 biological replicate). Statistical analyses were conducted with unpaired two-tailed *t* test: ns (not significant) *p* > 0.05, * *p* < 0.05, *** *p* < 0.001.

Hyperpolarized [1-^13^C] pyruvate has been widely applied for non-destructive assessment of the treatment response of cancer cells [17,37,38,39]. As demonstrated in our experiments above (Figure 3) and other studies [22,36], pyruvate-to-lactate flux strongly correlates with glycolytic flux, which is a central metabolic pathway. Therefore, it can be sensitively changed under stress conditions, including drug treatment. Our previous study with hyperpolarized [1-^13^C] pyruvate showed that the pyruvate-to-lactate flux in K562 cells (a leukemic cell line) substantially decreased after 24-h treatment with imatinib (tyrosine kinase inhibitor), whereas the cell number marginally decreased [22]. From that study, we found that NADH, a cofactor for pyruvate-to-lactate conversion by LDHA, decreased significantly with drug treatment, a direct reason for the decreased flux. Recently, ABT199 (venetoclax) in combination with hypomethylating agents has been used for leukemia treatment. ABT199 was shown to inhibit the electron transport chain complex in mitochondria and lower the NAD^+^ level [40,41,42] and increase the LDHA expression [43]. We hypothesized that ABT199 treatment would increase the pyruvate-to-lactate flux in the treated cells (Figure 3A). Consistent with previous studies [40,41,42,43], we confirmed that MOLM13 cells exhibited a lower level of NAD^+^ and a higher level of LDHA after 24-h treatment with 50 nM ABT199 (Figure 4A,B). We then assessed the pyruvate-to-lactate flux using the micro-slab coil with hyperpolarized [1-^13^C] pyruvate. MOLM13 cells treated with 0.1% DMSO (control group) or 50 nM ABT199 (treatment group) were loaded, and their fluxes were measured simultaneously. As we hypothesized, the pyruvate-to-lactate flux was higher in the treatment group (19.17 ± 3.12 pmol/s/10^5^ cells) than in the control group (6.37 ± 1.17 pmol/s/10^5^ cells) (Figure 4C). The levels of MCT1 and MCT4, other key factors contributing to the pyruvate-to-lactate flux, were not changed significantly with the treatment (Figure 4D and Figure S2B). Therefore, we reasoned that the pyruvate-to-lactate flux increase mainly resulted from the increase in the NADH/NAD^+^ ratio and LDHA level (Figure 4B,C). It is important to note that the cell number was markedly decreased only after 48-h treatment, while the cell viability was slightly decreased (Figure 4E,F). This indicates that the increased flux with drug treatment may be linked to the decreased proliferation, which can be explored in future studies.

## 4. Conclusions

This study demonstrates a novel micro-coil design that enables the sensitive detection of MR signals from multiple samples. Utilizing 3D printing technology, we built a multi-channel micro-slab coil that could provide a uniform sample-to-coil distance to multiple samples inside a coil while maintaining the detection sensitivity. This innovative platform effectively addressed the intrinsic limitation of the metabolic flux measurements using hyperpolarized probes. With further optimization, this micro-slab coil can accommodate more than three samples, notably increasing the throughput of hyperpolarized experiments for metabolic flux analysis. In addition, this coil design principle can be directly applicable to analysis of multiple samples sensitively in various MRI/MRS applications.

## Data Availability

The datasets supporting the conclusions of this article may be provided upon reasonable request.

## Supplementary Materials

The following supporting information can be downloaded at: https://www.mdpi.com/article/10.3390/bioengineering10010014/s1, Figure S1: Average distance between micro-solenoid and samples; Figure S2: Quantitative analysis of MCT1 and MCT4. Figure S3: Original western blot.
