# Supplementary material for: Micro-Slab Coil Design for Hyperpolarized Metabolic Flux Analysis in Multiple Samples
Source: Bioengineering (Basel). 2022 Dec 21;10(1):14. doi: 10.3390/bioengineering10010014 (PMC9854444; doi:10.3390/bioengineering10010014)
Supplement: Supplementary file 1 [file bioengineering-10-00014-s001.zip › bioengineering-2007884-supplementary.pdf]

bioengineering

MDPI

Supplementary Materials: Micro-Slab Coil Design for Hyperpolarized Metabolic Flux Analysis in Multiple Samples

Geonhui Lee [1,2,†], Thomas Ruan [3,4,5,†], Claudia Wong [1,2], Kofi Deh [3,4], Alli Abolarin [1,2], Alexander Correa [1], Kayvan R. Keshari [3,4,5,*] and Sangmoo Jeong [1,2,*]

Department of Chemical and Biomolecular Engineering, Johns Hopkins University, Baltimore, MD 21218, USA
[2] Institute for NanoBioTechnology, Johns Hopkins University, Baltimore, MD 21218, USA
[3] Department of Radiology, Memorial Sloan Kettering Cancer Center, New York, NY 10065, USA
[4] Molecular Pharmacology Program, Memorial Sloan Kettering Cancer Center, New York, NY 10065, USA
[5] Physiology, Biophysics and Systems Biology Program, Weill Cornell Medical College, New York, NY 10065, USA
* Correspondence: rahimikk@mskcc.org (K.R.K.) and sjeong@jhu.edu (S.J.)
† These authors contributed equally.

(A)

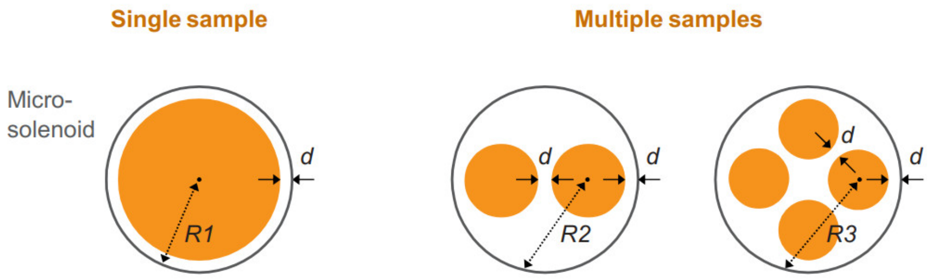

(B)

When '*d*' is	Average distance between micro-solenoid and center of sample(s)		
	One sample	*Two samples (R2)*	*Four samples (R3)*
$\frac{R1}{5}$	*R1*	*1.281 × R1*	*1.339 × R1*
$\frac{R1}{10}$	*R1*	*1.300 × R1*	*1.361 × R1*
$\frac{R1}{20}$	*R1*	*1.309 × R1*	*1.372 × R1*

Supplementary Figure S1. Average distance between micro-solenoid and samples. (A) Schematic of micro-solenoid and sample(s). (B) Calculated average distance between micro-solenoid and samples with different 'd'.

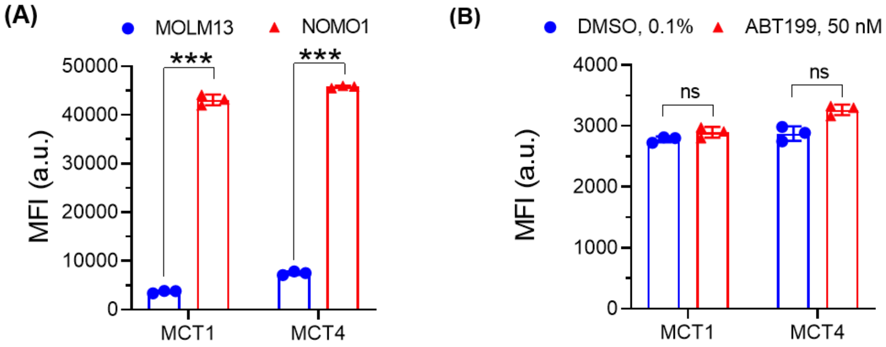

Supplementary Figure S2. Quantitative analysis of MCT1 and MCT4. (A) The surface expression levels of MCT1 and MCT4 in MOLM13 and NOMO1 cells. (B) The expression level of MCT1 and MCT4 in MOLM13 cells after 24-hr treatment of ABT199. Statistical analyses were conducted with unpaired two-tailed t test: *$p < 0.05$, **$p < 0.01$, ***$p < 0.001$.

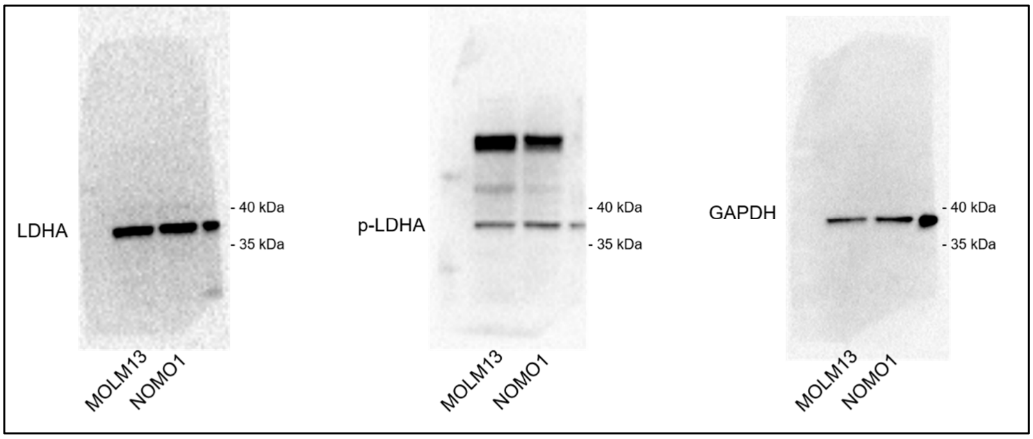

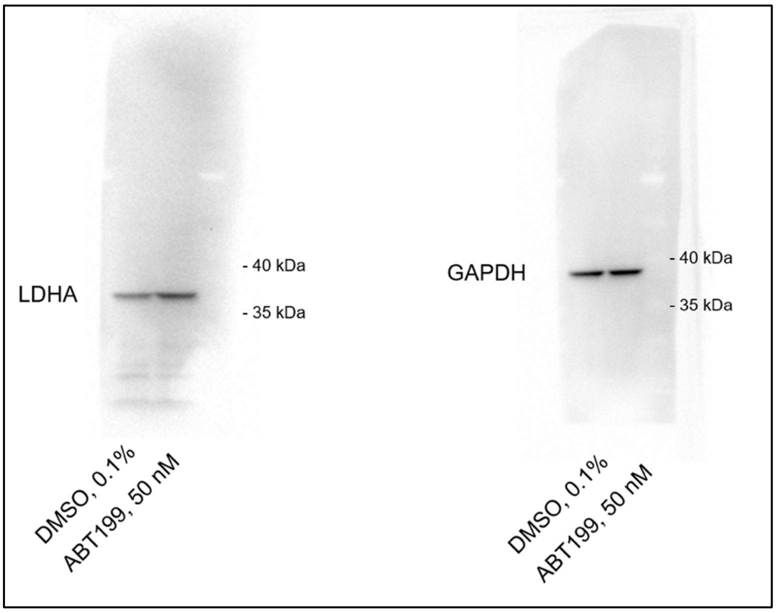

Supplementary Figure S3. Original western blot.
